# Soy isoflavones alleviate polycystic ovary syndrome in rats by regulating NF- κB signaling pathway

**DOI:** 10.1080/21655979.2021.1979864

**Published:** 2021-09-21

**Authors:** Xiaohong Ma, Xiaorong Li, LiLi Ma, Yan Chen, Shengyu He

**Affiliations:** a The Center for Reproductive Medicine, General Hospital of Ningxia Medical University, Yinchuan, China; b Department of Gynecology and Obstetrics, Shizuishan Central Hospital, the Fifth People’ S Hospital of Ningxia, Shizuishan City, China

**Keywords:** Soy isoflavones, letrozole, nf-κB signaling pathway, polycystic ovary syndrome

## Abstract

Soy isoflavones have been widely used in the treatment of clinical gynecological diseases. The aim of this study was to investigate the therapeutic effect and molecular mechanism of Soy isoflavones on rats with polycystic ovary syndrome (PCOS). Sprague-Dawley rats were orally administered 1 mg/kg letrozole for 21 consecutive days to induce the PCOS rat model. After PCOS induction, Soy isoflavones (100 mg/kg) or metformin (Positive control; 500 mg/kg) was administered continuously for 28 days. Then, H&E staining was used to observe the pathological changes of ovary. The serum hormone levels and the levels of antioxidant and inflammatory cytokines in ovarian tissue were detected. Additionally, the expression of NF-κB signaling pathway protein was detected by Western blot. Our results showed that soy isoflavones treatment significantly reduced the body weight, ovarian volume and weight, and improved estrous cycle in PCOS rats. H&E staining showed that the number of cystic dilated follicles and atretic follicles in ovarian tissue diminished, showing healthy follicles and corpus luteum. In addition, soy isoflavones treatment markedly decreased serum testosterone and luteinizing hormone (LH) levels, as well as oxidative stress levels and inflammation levels, and increased estradiol (E2) and follicle stimulating hormone (FSH) levels. At the same time, Soy isoflavones treatment inhibit the phosphorylation level of NF-κB p65 and increased the IκBα expression in ovarian tissues of PCOS rats. Overall, Soy isoflavones can improve ovarian morphology and hormone disorders in PCOS rats by inhibiting the activity of NF-κB pathway and enhancing anti-inflammatory and antioxidant capacity.

## 1. Introduction

Polycystic ovary syndrome (PCOS) is one of the most common reproductive endocrine and metabolic disorders in women of childbearing age. The main clinical symptoms are irregular menstruation, infertility, hyperandrogenemia, oligoovulation and polycystic ovary [1–3]. Meanwhile, it is also accompanied by metabolic abnormalities, such as obesity, insulin resistance, dyslipidemia and so on [4]. Some studies have discovered that the incidence of PCOS in women of childbearing age is about 5% to 10% [5]. On account of the etiology of PCOS is intricate, its clinical features are diverse and complex, which brings great pain to fertile women [6].

At present, PCOS is mainly treated with medications. Quite a few clinical trials have found that drug intervention in patients with PCOS can greatly improve the disorder of hormone levels and effectively relieve inflammation and other pathological features. It is reported that isoflavones are natural phytochemicals belonging to the category of phytoestrogens, which can provide potential alternative treatments for a variety of hormone-dependent diseases, such as cardiovascular diseases, female estrogen-like effects and anti-hormonal effects, bacteriostatic activity and prevention of osteoporosis and so on [7,8]. Soy isoflavones are a kind of mixture with polyphenol structure, which are separated and extracted from soy and are common phytoestrogens [9]. Several studies have shown that soy isoflavones change hormone levels by binding to estrogen receptors, thus regulating endocrine and metabolic disorders [10]. It also has the effect of aromatase inhibition and can affect the transformation of androgens to estrogens [11]. Current studies have shown that soy isoflavones have been used in the clinical treatment of PCOS patients [12]. Jamilian et al. [13] found that given soy isoflavones to women with PCOS for 12 weeks could prominently improve insulin resistance, hormonal status, and oxidative stress. Moreover, Rajan et al. [14] confirmed that soy isoflavones treatment reduced aromatase activity and testosterone concentration in PCOS rats. However, the pathogenesis of PCOS is more complex. Studies have demonstrated the ability of soy isoflavones to prevent and control cancer through anti-inflammatory and antioxidant effects [15]. But it is not clear whether the therapeutic effect of soy isoflavones on PCOS achieves efficacy through anti-inflammation. Therefore, in this study, letrazole-induced PCOS rat model was used to evaluate the therapeutic effect of soy isoflavones on PCOS from multiple aspects, such as estrous cycle, histopathological changes, inflammatory cytokines, antioxidant levels, hormone levels and NF-κB pathway, and analyze its possible mechanism.

## 2 Materials and methods

### 2.1 Animals

Healthy Sprague-Dawley female rats of Specific pathogen-free grade, aged 28 days, weighing 80 ± 5 g, were provided by the Experimental Animal Center of Reproductive Medicine Center of Ningxia Medical University General Hospital and provided with adaptive feeding for 14 days before the formal test. This experiment was approved by the Animal Ethics Committee of Reproductive Medicine Center of Ningxia Medical University General Hospital (Approval number: C202108-11).

### 2.2 Induction of PCOS model

Five of the 20 rats were randomly selected as the normal control group (oral administration of 1 mL normal saline for 28 days), and the remaining 15 rats were used to induce the PCOS model by giving 1 mg/kg letrozole for 21 consecutive days [16]. After successful PCOS induction, 15 PCOS rats were randomly and equally divided into three groups: the PCOS group (oral administration of 1 mL saline for 28 days), the MET group (oral administration of 1 mL saline dissolved with 500 mg/kg metformin for 28 days) [16] and the SI group (oral administration of 1 mL saline dissolved with 100 mg/kg soy isoflavones for 28 days) [14]. The body weight of rats in each group was measured weekly for the duration of the study. After the 7^th^ week (Day 49), the rats were sacrificed to measure the weight and the ovary of each rat was removed for weighing and measuring long and short diameters. And then the ovarian volume [mm^3^ = (π/6)*long (mm)*short (mm)^2^] was calculated as described by Wang [17]. Additionally, samples of ovarian and vaginal tissues and serum were collected for subsequent index detection. Letrozole was purchased from Jiangsu Hengrui Medicine Co., Ltd.; SI were purchased from Xi’an Tianfeng Biotechnology Co., Ltd.; and metformin was provided by Bristol-Myers Squibb Company.

### 2.3 Vaginal smears for Estrus cycle detection

The proportion of vaginal exfoliated cells was observed by vaginal tissue smears to judge the estrous cycle of rats. The vagina of rats was exposed, and cotton balls stained with normal saline were used to wipe the vulva area of rats to remove feces and other sundries. About 30 μL of normal saline was absorbed by the pipette, and the gun head of the pipette was placed in the vagina of the rats for repeated suction for 3–5 times. The saline was sucked out and dripped onto the prepared slides, whereupon the slides were placed under the light microscope to observe the variations of the estrous cycle of rats in each group. The judgment of period were as follows: leukocytes were staple in diestrus; non-keratinized nucleated epithelial cells were predominant in proestrus; anucleated keratinocytes were the majority in estrus, and the proportion of the three cells was comparable in metestrus [18].

### 2.4 Hematoxylin-eosin (H&E) staining

After the ovarian tissue was isolated, it was placed in formalin solution for fixation. The fixed ovarian tissues were routinely paraffin-embedded for sectioning and the slices thickness were 4 μm. After drying, the slices were placed in xylene for 10 min for deparaffinization and then put in different concentrations of ethanol for hydration. After being washed with distilled water for 2 min, the slices were stained by hematoxylin for 5–10 min. They were differentiated in 1% hydrochloric acid alcohol for 3 s and rinsed in tap water for 10–20 s. Two min of eosin staining later, they were dehydrated and transparent, and then sealed with neutral resin. The sections were observed using a microscope after drying.

### 2.5 Inflammation levels measurement

The ovarian tissues of rats were made into cell suspension. On the basis of the manufacturer’s instructions of enzyme-linked immunosorbent assay (ELISA) kit (Wuhan Huamei Bioengineering Co., Ltd., China), the tissue was diluted to an appropriate concentration. The reagent and sample were added to the ELISA plate in sequence by ELISA. After the reaction was completed, the ELISA plate was placed at 450 nm wavelength to detect the optical density (OD). The levels of TNF-α, IL-1β, and IL-6 in ovarian tissue were calculated according to the standard curve.

### 2.6 Hormone and oxidative stress levels measurement

The ovarian tissue was ground into tissue homogenate by tissue grinder, and the supernatant was prepared for later use after centrifugation. According to the instructions of the kit, the serum and the supernatant of tissue homogenate were diluted into different concentration gradients standby liquid. The 96-well ELISA plate was placed under 550, 412, 532 nm wavelengths to detect the OD. According to the standard curve, the activities of superoxide dismutase (SOD), glutathione peroxidase (GPx) and the content of malondialdehyde (MDA) in serum and ovarian tissue were converted. Then, the 96-well ELISA plate was placed at 450 nm wavelength to detect the OD, according to the standard curve, the levels of testosterone (T), estradiol (E2), luteinizing hormone (LH) and follicle stimulating hormone (FSH) and the LH/FSH ratio was calculated.

### 2.7 Western blot

The ovarian tissues of rats were lysed with RIPA lysate. The protein was collected and the protein concentration was determined by BCA protein assay reagent (Biyuntian, China). The protein was then separated by 10% SDS-PAGE and transferred to the PVDF membrane (Merck Millipore, Germany). After blocking with the skimmed milk at room temperature for 1 h, the membrane was then incubated at 4°C overnight with primary antibody NF-κB p65, p-NF-κB p65, IκBα, p-IκBα (all form Cell Signaling Technology, USA) and β-actin (Proteintech, USA). The membrane was washed twice, then diluted enzyme-labeled second antibody was added and incubated at room temperature for 1 h. The protein level was analyzed with β-actin as the internal reference.

### 2.8 Statistical analysis

All experimental data were statistically analyzed by SPSS version 22.0 software. Student’s *t*-test was used between the two groups, and one-way analysis of variance (ANOVA) was used for comparison between multiple groups. Results were expressed as mean ± standard deviation (SD) and P < 0.05 was recorded as statistically significant.

## 3 Result

### 3.1 Effects of soy isoflavones on body weight and ovarian weight of PCOS rats

**Figure 1.**
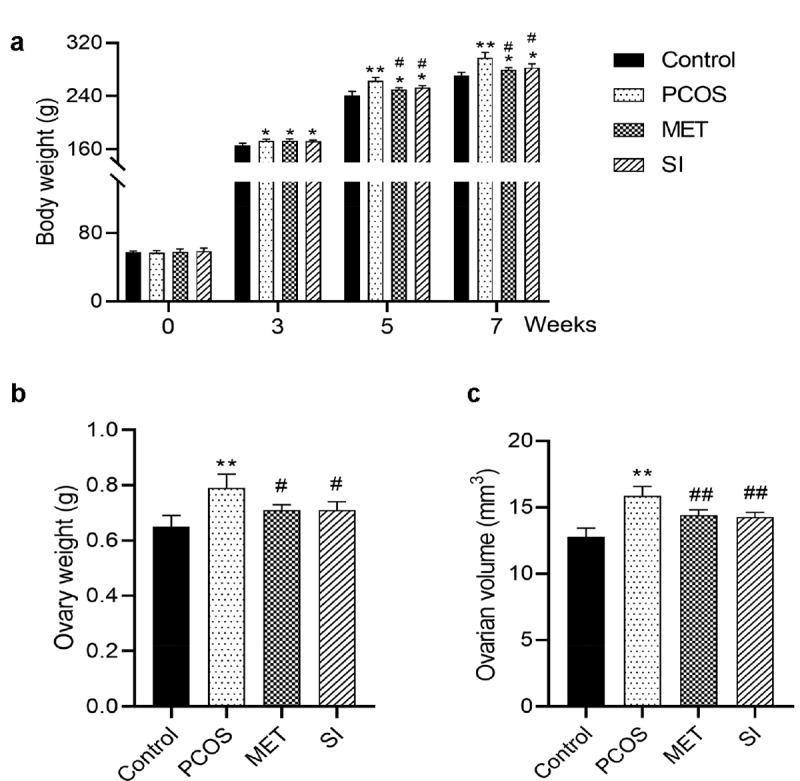
Effects of soy isoflavones on body weight and ovarian weight of PCOS rats. A, Changes of body weight of rats in each group; B-C, Changes of ovarian weight and volume of rats in each group after 4 weeks of soy isoflavones treatment in PCOS rats. * P < 0.05 and ** P < 0.01 vs. Control group; ^#^ P < 0.05 and ^##^ P < 0.01 vs. PCOS group. PCOS, Polycystic ovary syndrome; MET, Metformin; SI, soy isoflavones

Compared with the Control group, the body weight of rats in each group increased significantly after 3 weeks of letrozole administration induction (P < 0.05). At the 5^th^ and 7^th^ week, the body weight of rats in MET group and SI group was significantly lower than that in PCOS group (Figure 1(a)). After week 4 of soy isoflavones treatment in PCOS rats, the weight and volume of rat ovaries were significantly reduced (P < 0.05) (Reduced by 10.1% and 9.9%, respectively; Figure 1(b-c)). Metformin-treated PCOS rats showed the same results as the soy isoflavones group.

### 3.2 Effects of soy isoflavones on estrus cycles of PCOS rats

**Figure 2.**
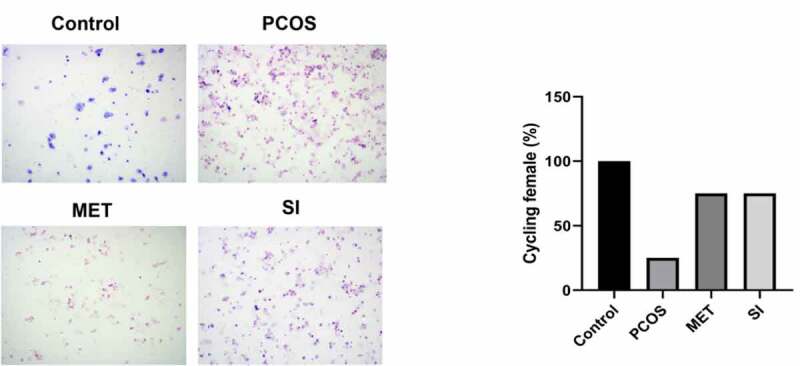
Effect of soy isoflavones on estrus cycle of PCOS rats PCOS, polycystic ovary syndrome; MET, Metformin; SI, soy isoflavones

To evaluate the effect of soy isoflavones on the estrus cycle in rats, we used vaginal smear to verify the stages of the estrus cycle. Figure 2 showed that compared with the Control group, the estrus cycle of rats in the PCOS group was almost irregular. However, after treatment with Metformin and soy isoflavones, the population of PCSO rats with estrus cycles showed recovery up to 75%.

### 3.3 Effects of soy isoflavones on ovarian histological morphology of PCOS rats

**Figure 3.**
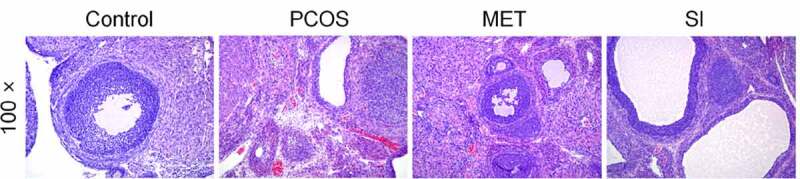
Effect of soy isoflavones on ovarian histological morphology of PCOS rats Magnification, 100 × . PCOS, Polycystic ovary syndrome; MET, Metformin; SI, soy isoflavones

The morphological changes of ovaries are the main characteristics of patients with PCOS. We observed the histological changes of ovaries by H&E staining. As shown in Figure 3, contrasted with Control group, the ovarian tissues of the rats in the PCOS group had more atretic follicles, fewer granulosa cell layer, hyperplasia of the theca layer, and corpus luteum was scarcely found. In comparison with PCOS group, the number of atretic follicles in MET and SI groups are less than Control group, while the granulosa cell layer increased, and healthy follicles and corpus luteum could be seen.

#### 3.4 Effects of soy isoflavones on serum hormone levels of PCOS rats

To further investigate whether SI can regulate hormone levels of PCOS rats.

**Figure 4.**
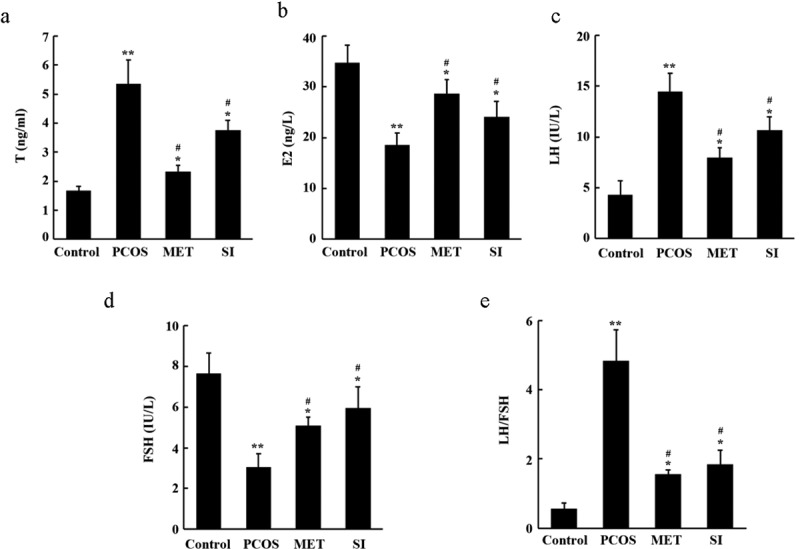
Effects of soy isoflavones on serum hormone levels of PCOS rats. A, Biochemical test of serum testosterone (T) levels of rats; B, Biochemical test of serum estradiol hormone (E2) levels of rats; C, Biochemical test of serum luteinizing hormone (LH) levels of rats; D, Biochemical test of the levels of follicle-stimulating hormone (FSH) in serum of rats; E, LH/FSH ratio.* P < 0.05 and ** P < 0.01 vs. Control group; ^#^ P < 0.05 vs. PCOS group. PCOS, Polycystic ovary syndrome; MET, Metformin; SI, soy isoflavones

As shown in (Figure 4(a-e)), compared with Control group, the serum T, LH levels, and LH/FSH ratio increased significantly, while the E2 and FSH levels declined notably in the PCOS group (P < 0.05). But contrasted with the PCOS group, MET and SI reduced the serum T, LH levels, and LH/FSH ratio, raised E2 and FSH levels significantly (P < 0.05). There was no significant difference between MET group and SI group (P > 0.05).

### 3.5 Effects of soy isoflavones on oxidative stress and inflammatory cytokines in PCOS rats

**Figure 5.**
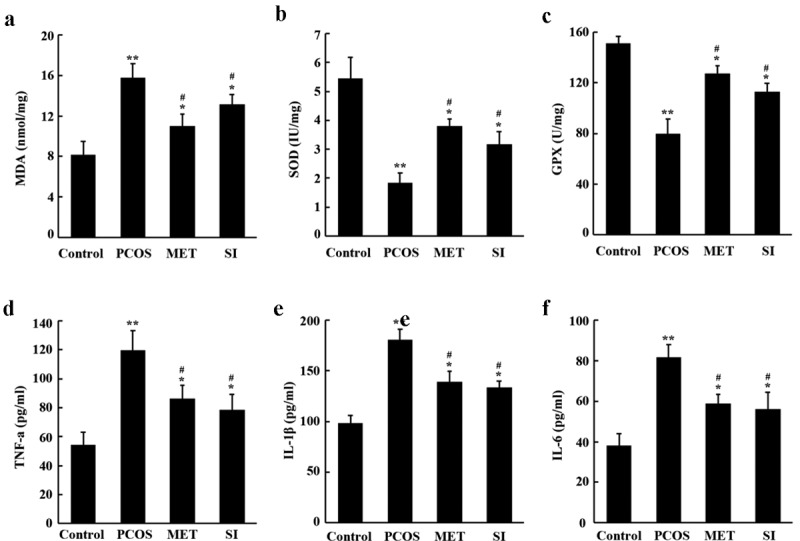
Effects of soy isoflavones on oxidative stress and inflammatory cytokines in ovarian tissues of PCOS rats. A-C, Biochemical detection of MDA, SOD and GPX levels in rat ovarian tissue; D-F, ELISA determination of the levels of TNF-α, IL-1β and IL-6 in ovarian tissue of rats. *P < 0.05 the and * * P < 0.01 vs Control group; ^#^P < 0.05 vs. PCOS group. PCOS, Polycystic ovary syndrome; MET, Metformin; SI, soy isoflavones

Inflammation and oxidative stress are considered to be an important pathological process of PCOS [19,20]. (Figure 5(a-c)) showed that in comparison with the Control group, the activity of MDA in ovarian tissue of each group was significantly improved, while the activity of SOD and GPx decreased (P < 0.05). Compared with the PCOS group, MET and SI could reverse the expression of oxidative stress level in PCOS rats (P < 0.05). The results of 5D-F showed that the levels of TNF-α, IL-1β and IL-6 in ovarian tissue of rats in each group were obviously higher than those in the Control group. However, MET and SI treatment groups significantly reduced the levels of TNF-α, IL-1β, and IL-6 in PCOS rats (P < 0.05).

#### 3.6 Effect of soy isoflavones on NF-κB signaling pathway in ovarian tissue of PCOS rats

**Figure 6.**
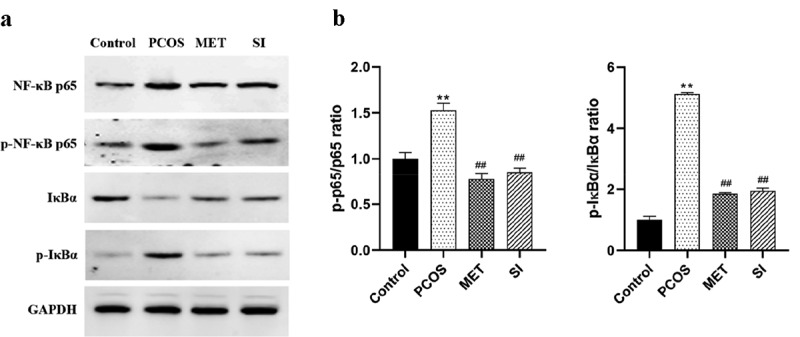
Effect of soy isoflavones on NF-κB signaling pathway in ovarian tissue of PCOS rats

NF-κB is regarded as the key mediator of inflammatory process [21]. The results of Western blot (Figure 6(a-b)) showed that compared with the Control group, the ratio of p-p65/p65 and p-IκBα/IκBα in PCOS group increased significantly (P < 0.05). Compared with the PCOS group, the ratio of p-p65/p65 and p-IκBα/IκBα in ovarian tissues of the MET group and the SI group were significantly reduced (P < 0.05). And there was no significant difference between MET group and SI group (P > 0.05).

A, Western blot was used to detect the expression of NF-κB signaling pathway-related proteins in the ovarian tissues of rats in each group; B, Statistical map of protein grayscale analysis. **P < 0.01 vs. Control group; ^#^P < 0.05 vs. PCOS group. PCOS, Polycystic ovary syndrome; MET, Metformin; SI, soy isoflavones.

## 4 Discussion

PCOS is a kind of endocrine and metabolic disorder syndrome with diverse and complex causes and highly specific clinical manifestations [22]. In recent years, modern medicine has regarded PCOS as a subclinical inflammatory response, which damages insulin B cells, and then damages the ovary leading to abnormal hormone secretion and endocrine disorders [23–25]. Therefore, the treatment of PCOS has become a hotspot of research in reproductive diseases. The 2018 edition of ‘Guidelines for the Diagnosis and Treatment of Polycystic Ovarian Syndrome in China’ clearly points out that metformin can be added or used alone in the treatment of PCOS patients [26]. Therefore, in this study, we used metformin as the positive control drug to explore the possible molecular mechanism of SI in the treatment of PCOS.

In this study, we found that SI can reduce body weight, ovarian volume and weight and improve irregular estrus in PCOS rats, which is consistent with the results of Yan et al. [27]. In addition, this study also observed that SI had a regulatory effect on PCOS hormone disorder. After SI intervention, serum T and LH levels were down-regulated, the ratio of LH/FSH was decreased, and E2 and FSH levels were significantly evaluated in PCOS rats. This suggests that the hormone disorder of PCOS rats has been improved. Abnormal elevation of LH secretion is the main feature of PCOS [28], which can induce androgen biosynthesis through endometrial cells and increase androgen levels in the body [3]. After the intervention of SI, the expression of serum LH decreased and androgen biosynthesis was down-regulated, which was beneficial to reduce the level of androgen in PCOS rats. This animal model is made with letrozole, which is characterized by hyperandrogenic and hypoestrogenic states. SI exert estrogen-like effects after intervention, supplement estrogen in the body to counteract the excessive synthesis of androgen, and reduce the level of serum T; at the same time, SI also have the effects of aromatase inhibitor, which promote FSH secretion by blocking the conversion of androgens to estrogens and negative feedback regulation, and then induce ovulation [29]. Several studies have shown that PCOS patients have increased levels of oxidative stress represented by MDA, lipid peroxides, and protein carbonyls, and decreased antioxidant levels represented by retinol, superoxide dismutase, and total antioxidant capacity [30,31]. Abnormalities in oxidative stress response in PCOS patients are involved in the pathological process of insulin resistance, hyperandrogenism formation, and also play an important role in oocyte maturation and embryonic development [32]. In this study, we found that SI notably lowered the activity of MDA and increased the activity of SOD, GPx, indicating that SI have antioxidant effect. As a key transcription factor in the initiation and regulation of inflammation, NF-κB plays an important role in inflammatory response. NF-κB mediated inflammatory signaling pathway is a classical signaling pathway for the regulation of inflammatory response. In the resting state, NF-κB protein binds with its inhibitor IkBα to form inactive trimer. When cells are stimulated by intracellular and exogenous sources, IkBα will undergo phosphorylation, ubiquitination and degradation, release NF-κB translocation to the nucleus and phosphorylation. By combining with transcriptional regulatory elements, it activates the transcription of multiple downstream inflammatory genes, thus triggering inflammation and releasing inflammatory factors [33]. The results of this study showed that the expression levels of NF-κB p65, p-NF-κB p65 and p-IκBα in ovarian tissues of PCOS rats were risen significantly, while the expression of IκBα was prominently decreased, and the levels of inflammatory cytokines TNF-α, IL-1β, and IL-6 were also remarkably increased. However, after the intervention if SI, the related expression was reversed. These results suggest that PCOS rats present a state of chronic proliferative inflammation, and the intervention of SI can inhibit the NF-κB inflammatory signaling pathway, thereby reducing the release of downstream inflammatory factors and improving the inflammatory state.

## 5 Conclusion

In summary, in letrozole-induced PCOS rats, soy isoflavones may reduce the release of inflammatory cytokines by inhibiting the activation of NF-κB signaling pathway, thereby regulating serum hormone disorders and improving the morphology and function of polycystic ovaries. Additionally, they also enhance the antioxidant capacity of rats, which provides an experimental basis for the application of SI in the clinical treatment of PCOS. However, further studies are needed on how soy isoflavones inhibit the activation of NF-κB signaling pathway.

## HIGHLIGHTS

1. Soy isoflavones treatment reduces the body weight, ovarian volume and weight, and improved estrous cycle in PCOS rats.
2. Soy isoflavones treatment decreases serum testosterone levels and increased E2 and FSH levels in PCOS rats.
3. Soy isoflavones treatment reduces oxidative stress levels and inflammation levels in PCOS rats.
4. Soy isoflavones treatment inhibits the activity of NF-κB pathway in PCOS rats.
